# Using Asthma-Related Housing Complaints to Target Residents With Uncontrolled Asthma in Salt Lake County, Utah

**DOI:** 10.5888/pcd16.180463

**Published:** 2019-05-23

**Authors:** Jiyoung Byun, Sophie McDonnell, Jenny Robertson

**Affiliations:** 1 Epidemiology Bureau, Salt Lake County Health Department, Salt Lake City, Utah; 2 Health Promotion Bureau, Salt Lake County Health Department, West Jordan, Utah

**Figure:**
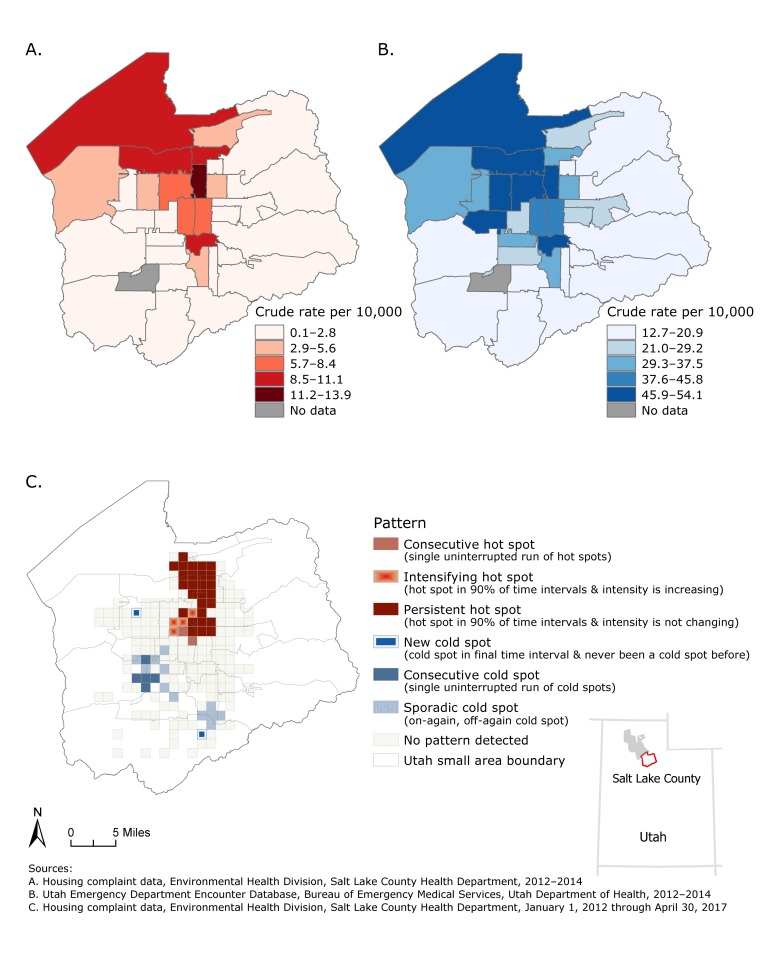
Maps A and B compare rates of asthma-related housing complaints and rates of asthma-related emergency department encounters, by small-area boundaries, Salt Lake County, Utah, 2012–2014. Map C depicts hot spots of asthma-related housing complaints that were identified in north-central Salt Lake County, Utah, January 1, 2012, through April 30, 2017.

## Background

The prevalence of asthma is high in Salt Lake County, Utah; 9.5% of adults aged 18 years or older (1) and 6.7% of children and adolescents aged 0 to 17 years have asthma (2). On average, 1,800 adults and 1,200 children visit an emergency department (ED) (3) and 400 adults and 400 children are hospitalized (4) with a primary diagnosis of asthma each year.

In 2015, the Utah Asthma Program and partners from the Utah Asthma Task Force developed the Utah Asthma Home Visiting Program (UAHVP). This program serves families with uncontrolled asthma and is only available in Salt Lake and Utah counties (5). The Salt Lake County Health Department (SLCoHD) collaborated with the Utah Asthma Program to explore using recent and up-to-date housing complaint data to more efficiently target the UAHVP. Currently, the UAHVP is targeted in areas by using ED data, which have a reporting lag time of several years. In comparison, housing complaint data are collected in real time and readily accessible from the SLCoHD Environmental Health Division. 

The goals of this project were to retrospectively identify asthma-related housing complaints, geocode these complaints, assess their relationship to the rate of asthma ED encounters, and analyze emerging hot spots to determine whether these data and methods could identify communities that may benefit from the UAHVP.

## Methods

Housing complaints reported to the SLCoHD Environmental Health Division from January 1, 2012, to April 30, 2017, were manually reviewed and categorized as asthma-related if the complaint described an asthma trigger defined by the Centers for Disease Control and Prevention (CDC) (eg, smoke, mites, mold, pets, cockroaches, rodents, strong odors, cigarettes, birds, pollution) (6). The final data set included 1,959 asthma-related housing complaints. Geocoding and spatial analyses were performed by using ArcGIS Pro 2.0 (Esri). Ninety-nine percent of complaints were geocoded with a match score of 90 or higher, aggregated to a small area as defined by the Utah Department of Health (7), and used to calculate and map crude incidence rates.

Crude rates of asthma ED encounters from 2012 through 2014 were mapped by Utah small area and compared visually with crude rates of asthma-related housing complaints from 2012 through 2014 (8). The Pearson correlation coefficient between rates was calculated by using Microsoft Excel (Microsoft Corp) to determine the strength of the relationship.

We analyzed emerging hot spots (9) of asthma-related housing complaints by aggregating cases into space-time cubes of 6 months and 5,500 feet and evaluating trends over time by using a neighborhood distance of 11,000 feet and a time-step interval of 2. The appropriate distance band for the space-time cube was determined by plotting global Moran’s *I* *z*-scores from spatial autocorrelation analysis using 1,000-foot intervals from 1,000 to 20,000 feet and identifying the distance with the highest *z*-score peak (5,500 feet).

## Findings

Visual comparison suggested that the rate of asthma-related housing complaints was positively correlated with the rate of asthma ED encounters by small area. Correlation analysis supported this finding and indicated a strong positive relationship (*r* = 0.77). Analysis of emerging hot spots of asthma-related housing complaints identified consecutive, intensifying, and persistent hot spots in communities of north central Salt Lake County. These hot spots may reflect communities with older housing that may benefit from the resources provided by the UAHVP.

## Action

Our findings demonstrate the potential of using asthma-related housing complaints as a current, proxy data source for measuring asthma burden and of analyzing emerging hot spots to target or expand the UAHVP. Next steps include investigating factors that explain the spatial pattern of asthma-related housing complaints. If the pattern can be explained by factors addressed in the UAHVP or by participating partners, such as Green and Healthy Homes, a national initiative to create safe and healthy homes for low-income families, the findings would provide additional support for the use of these data and methods to guide program decisions. Further exploratory work could investigate the types, number, and causes of asthma triggers occurring in hot spots, which could be useful for measuring severity and customizing asthma control strategies in neighborhoods.

This project had several limitations. First, we could not confirm that the positive relationship of asthma-related housing complaints with asthma ED encounters existed in recent years because we lacked recent data on ED encounters. Second, housing complaints are reported predominantly by renters, so asthma-related housing issues that may exist for homeowners were not captured. Third, the high rates of asthma ED encounters were likely influenced by the underlying spatial distribution of social determinants that contribute to asthma burden, such as low household income and barriers to health care access.
